# Association between *apurinic/apyrimidinic endonuclease 1* rs1760944 T>G polymorphism and susceptibility of cancer: a meta-analysis involving 21764 subjects

**DOI:** 10.1042/BSR20190866

**Published:** 2019-12-17

**Authors:** Guowen Ding, Yu Chen, Huiwen Pan, Hao Qiu, Weifeng Tang, Shuchen Chen

**Affiliations:** 1 Department of Cardiothoracic Surgery, Affiliated People’s Hospital of Jiangsu University, Zhenjiang, Jiangsu Province, China; 2 Department of Medical Oncology, Fujian Cancer Hospital, Fujian Medical University Cancer Hospital, Fuzhou, Fujian Province, China; 3 Department of Immunology, School of Medicine, Jiangsu University, Zhenjiang, Jiangsu Province, China; 4 Department of Thoracic Surgery, Fujian Medical University Union Hospital, Fuzhou, Fujian Province, China

**Keywords:** APE1, Cancer, Meta-analysis, Polymorphism

## Abstract

**Background:** Previous case–control studies have suggested that *apurinic/apyrimidinic endonuclease 1* (*APE1*) rs1760944 T>G polymorphism may be associated with cancer risk. Here, we carried out an updated meta-analysis to focus on the correlation between *APE1* rs1760944 T>G locus and the risk of cancer.

**Methods:** We used the crude odds ratios (ORs) with their 95% confidence intervals (CIs) to evaluate the possible relationship between the *APE1* rs1760944 T>G polymorphism and cancer risk. Heterogeneity, publication bias and sensitivity analysis were also harnessed to check the potential bias of the present study.

**Results:** Twenty-three independent studies involving 10166 cancer cases and 11598 controls were eligible for this pooled analysis. We found that *APE1* rs1760944 T>G polymorphism decreased the risk of cancer in four genetic models (G vs. T: OR, 0.87; 95% CI, 0.83–0.92; *P*<0.001; GG vs. TT: OR, 0.77; 95% CI, 0.69–0.86; *P*<0.001; GG/TG vs. TT: OR, 0.83; 95% CI, 0.77–0.89, *P*<0.001 and GG vs. TT/TG: OR, 0.85; 95% CI, 0.80–0.92, *P*<0.001). Results of subgroup analyses also demonstrated that this single-nucleotide polymorphism (SNP) modified the risk among lung cancer, breast cancer, osteosarcoma, and Asians. Evidence of publication bias was found in the present study. When we treated the publication bias with ‘trim-and-fill’ method, the adjusted ORs and CIs were not significantly changed.

**Conclusion:** In conclusion, current evidence highlights that the *APE1* rs1760944 T>G polymorphism is a protective factor for cancer susceptibility. In the future, case–control studies with detailed risk factors are needed to confirm or refute our findings.

## Introduction

The incidence and mortality of cancer is increasing worldwide [1–3]. It was estimated that approximately 18.1 million new cancer patients were diagnosed and more than half of them died worldwide during 2018 [1]. The etiology of cancer is complicated. Previous epidemiological studies have indicated that consumption of red meat, fried and salted meat, tobacco smoking and alcohol abuse, diabetes mellitus, obesity, non-alcoholic fatty liver disease, oxidative stress, chronic infection and inflammation can contribute to the development of cancer [4–6]. However, these potential risk factors could not fully explain the etiology of cancer. It is reported that the hereditary factor may influence the susceptibility of cancer [7,8].

Apurinic/apyrimidinic endonuclease 1 (APE1) is a multifunctional protein which plays an important role in the pathway of base excision repair (BER). APE1 plays a pivotal role in tumor cells involving DNA damage response and regulating transcription factor activation [9]. The observed roles of APE1 protein allude to its potential effect on inflammation, growth, migration and angiogenesis [9–11]. In addition, APE1 may be also implicated in regulating cell cycle, oxidative stress and apoptosis [12]. Recently, some investigations reported that the expression level of APE1 was up-regulated in a number of cancers [13–17]. In addition, glioma cell with higher APE1 expression level was also associated with shorter time to tumor progression after chemo/radiotherapy [18,19]. As well, previous studies reported that the decreased APE1 activity might retard cell growth of ovarian cancer [20] and pancreatic cancer [21].

*APE1* gene is approximately 3 kb in length and is located on chromosome 14q11.2 [22]. A number of variants in *APE1* gene are established (https://www.ncbi.nlm.nih.gov/snp/?term=APE1). *APE1* rs1760944 (−656T>G) is a promoter locus and has been widely explored. Some functional studies indicated that the *APE1* rs1760944 T>G single-nucleotide polymorphism (SNP) might decrease APE1 mRNA and protein expression levels [23,24]. Many case–control studies were conducted to identify the potential association of *APE1* rs1760944 T>G polymorphism with the development of cancer. Individuals with *APE1* rs1760944 GG variant might reduce 46% glioblastoma risk than those who carried *APE1* rs1760944 TT variant [25]. The relationship between *APE1* rs1760944 T>G polymorphism and a decreased susceptibility of lung cancer was also found by Lu et al. [24]. A previous study reported that gastric cancer cases carried *APE1* rs1760944 GT/GG variants might have a better survival than others with *APE1* rs1760944 TT genotype [26]. But the results were conflicting. Two meta-analyses suggested that this SNP was correlated with a decreased susceptibility of cancer in Asian populations and lung cancer [27,28]. Recently, many investigations focused on the association between *APE1* rs1760944 T>G polymorphism and the risk of other cancers. The findings were more confusing. The aim of the present study was to carry out a meta-analysis to evaluate whether this SNP was associated with the risk of cancer.

## Materials and methods

### Literature search parameters

PubMed and Embase databases were exhaustively searched for relevant publications which studied the relationship of *APE1* rs1760944 T>G locus with the risk of cancer from the inception up to 17 March 2019. The search strategy was: (polymorphism OR SNP) and (apurinic/apyrimidinic endonuclease 1 or APE1 or APE-1) and (cancer OR carcinoma). In the current study, publications written in English or Chinese were eligible. Moreover, the references of the included studies, comments, meta-analyses and reviews were manually retrospected to recruit the potential literatures.

### Inclusion criterion

For eligibility, publications were required to meet the following inclusion criteria: (1) case–control studies investigating the relationship between the *APE1* rs1760944 T>G locus and the risk of cancer; (2) the diagnosis of cases was confirmed by pathological examination; (3) the frequencies of alleles or genotypes were presented; (4) the paper was written in English or Chinese.

### Exclusion criteria

Studies were excluded based on the major exclusion criteria: (1) not case–control design; (2) studies did not provide genotyping data on *APE1* rs1760944 T>G polymorphism; and (3) meta-analyses/reviews, comments and letters focusing on the relationships between the *APE1* rs1760944 T>G locus and cancer risk.

### Data extraction

Two authors (Guowen Ding and Yu Chen) reviewed each eligible study independently. They extracted the following terms from case–control studies, including the first author name, publishing year, country where the study was carried out, ethnicity, the source of control, cancer type, numbers of included cases and controls in each case–control study, genotyping data, the method of polymerase chain reaction, statistical method and evidence of Hardy–Weinberg equilibrium (HWE) evaluation in control group. If the extracted data had any dispute, authors settled these issues following a detailed discussion among all reviewers.

### Statistical analysis

HWE in controls was assessed by an online Pearson’s χ^2^ test (http://ihg.gsf.de/cgi-bin/hw/hwa1.pl). We calculated crude odds ratios (ORs) and 95% confidence intervals (CIs) to evaluate the correlation of *APE1* rs1760944 T>G polymorphism and cancer risk. The following four genetic models were used, including homozygote model (GG vs. TT), dominant model (GG/TG vs. TT), recessive model (GG vs. TT/TG) and allele model (G vs. T). Cochran’s Q-statistic and *I^2^* test were used to check the heterogeneity among the included studies. The random-effect model was harnessed when *I^2^* > 50% or *P*<0.10 [29]; otherwise, a fixed-effect model was used [30]. Subgroup analyses were performed to explore the heterogeneity source among the studies. Ethnicity, the source of control and cancer type was considered as the potential source of heterogeneity. Begg’s funnel plots and Egger’s linear regression test were used to detect the potential bias in this meta-analysis. Since significant bias was identified in the present study, non-parametric ‘trim-and-fill’ method was used to evaluate the stability of the observed results. Sensitivity analysis was conducted by one-way method, which deleted each study one by one and re-calculated the pooled ORs and CIs. All statistical analyses were conducted by using STATA 12.0 (Stata Corporation, TX, U.S.A.). A *P*-value (two-sided) <0.05 was defined as statistically significant.

## Results

### Characteristics of eligible case–control studies

**Figure 1:**
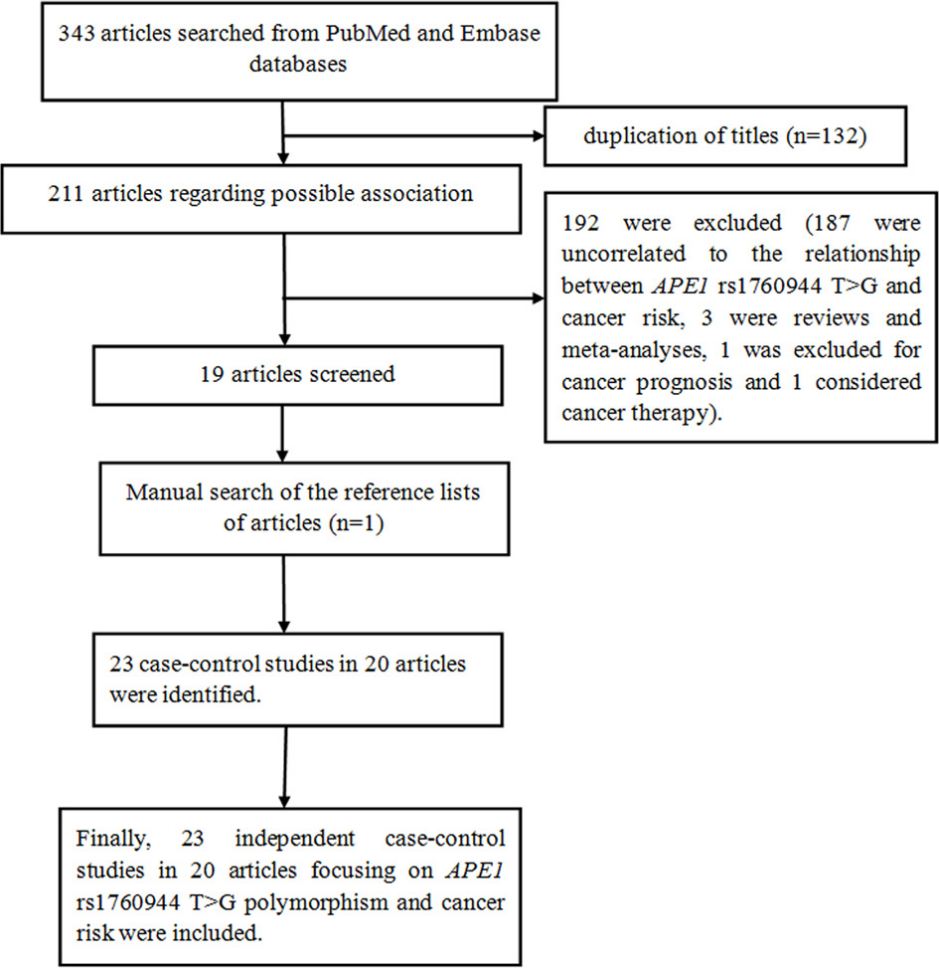
Flow diagram of included and excluded processes

Figure 1 shows the selection process of the eligible publications. A total of 343 papers were collected. According to the major inclusion criteria, there were 20 papers (including 23 independent case–control studies) focusing on the relationship of *APE1* rs1760944 T>G polymorphism with cancer risk [23–25,31–47]. Among them, five investigated lung cancer [24,31–33], three investigated colorectal cancer [34–36], three investigated breast cancer [37–39], three investigated cervical cancer [40,41], two investigated osteosarcoma [23], two investigated nasopharyngeal carcinoma [42,43] and five investigated other cancers (bladder cancer [44], glioblastoma [25], renal cell carcinoma [45], prostate cancer [46] and ovarian cancer [47]). Additionally, twenty-one had Asian and two had Caucasian ethnicities. In all included studies, χ^2^ test was used to calculate the pooled ORs and CIs. The detailed characteristics of the included case–control studies are shown in Table 1. The number of each genotype and HWE are presented in Table 2.

**Table 1:** Characteristics of all included studies in the meta-analysis

Author	Year	Country	Ethnicity	The type of cancer	Genotyping method	Source of control	Sample size (case/control)	Statistical methods
Berndt et al.	2007	U.S.A.	Caucasians	Advanced colorectal adenoma	Taqman	PB	767/720	χ^2^ test
Lu et al.	2009	China	Asians	Lung cancer	Illumina	PB	500/517	χ^2^ test, SPSS 15.0
Lo et al.	2009	China	Asians	Lung cancer	MassARRAY	HB	730/730	χ^2^ test, SAS
Lu et al.	2009	China	Asians	Lung cancer	Illumina	HB	572/547	χ^2^ test, SPSS 15.0
Wang et al.	2010	China	Asians	Bladder cancer	PCR-RFLP	HB	234/253	χ^2^ test, SAS
Zhou et al.	2011	China	Asians	Glioblastoma	MALDI-TOF	HB	766/824	χ^2^ test, SPSS 15.0
Li et al.	2011	China	Asians	Lung cancer	PCR-CTPP	HB	455/443	χ^2^ test, SPSS 16.0
Cao et al.	2011	China	Asians	Renal cell carcinoma	TaqMan	HB	612/632	χ^2^ test, *t* test, SAS
Wang et al.	2013	China	Asians	Cervical cancer	PCR-RFLP	HB	306/306	χ^2^ test, *t* test, SAS
Jing et al.	2013	China	Asians	Prostate cancer	PCR-RFLP	HB	198/156	χ^2^ test, SPSS 16.0
Kang et al.	2013	China	Asians	Breast cancer	TaqMan	HB	500/799	χ^2^ test, SAS
Pan et al.	2013	China	Asians	Lung cancer	PCR-LDR	HB	819/803	χ^2^ test, Open-source R software
Zhang et al.	2013	China	Asians	Ovarian cancer	DNA sequence	HB	124/141	χ^2^ test, SPSS 16.0
Li et al.	2013	China	Asians	Nasopharyngeal carcinoma	PCR-CTPP	HB	231/300	χ^2^ test, SPSS 16.0
Zhang et al.	2014	China	Asians	Colorectal cancer	PCR-CTPP	HB	247/300	χ^2^ test, SPSS 19.0
Luo et al.	2014	China	Asians	Breast cancer	PCR-CTPP	HB	194/245	χ^2^ test, SPSS 16.0
Mashayekhi et al.	2015	Iran	Caucasians	Breast cancer	T-ARMS-PCR	HB	150/150	χ^2^ test, Medcalc software 12.1
Lai et al.	2016	China	Asians	Colorectal cancer	High resolution melting assay	HB	727/736	χ^2^ test, SAS9.2
Meng et al.	2017	China	Asians	Cervical cancer	TaqMan	HB	571/657	χ^2^ test
Meng et al.	2017	China	Asians	Cervical cancer	TaqMan	HB	608/1165	χ^2^ test
Xiao et al.	2017	China	Asians	Osteosarcoma	TaqMan	HB	172/256	χ^2^ test, SPSS 22.0, GraphPad Prism 6.0
Xiao et al.	2017	China	Asians	Osteosarcoma	TaqMan	HB	206/360	χ^2^ test, SPSS 22.0, GraphPad Prism 6.0
Lu et al.	2017	China	Asians	Nasopharyngeal carcinoma	MassARRAY	HB	477/558	χ^2^ test, SPSS 17.0

Abbreviations: MALDI-TOF MS, matrix-assisted laser desorption/ionization time of flight mass spectrometry; PCR-CTPP, polymerase chain reaction with confronting two-pair primers; PCR-LDR, polymerase chain reaction-ligase detection reaction; PCR-RFLP: polymerase chain reaction-restriction fragment length polymorphism; T-ARMS-PCR, tetra-primer amplification refractory mutation system-polymerase chain reaction.

**Table 2:** Distribution of *APE1* rs1760944 T>G polymorphism genotype and allele among cases and controls

Author	Year	case			control			case		control		HWE
		TT	TG	GG	TT	TG	GG	G	T	G	T	
Berndt et al.	2007	106	310	244	114	317	243	798	522	803	545	Yes
Lu et al.	2009	184	241	75	170	238	109	391	609	456	578	Yes
Lo et al.	2009	271	332	122	234	341	153	576	874	647	809	Yes
Lu et al.	2009	199	288	85	149	293	105	458	686	503	591	Yes
Wang et al.	2010	92	108	34	77	124	52	176	292	228	278	Yes
Zhou et al.	2011	233	392	125	237	424	155	642	858	734	898	Yes
Li et al.	2011	162	227	66	143	206	94	359	551	394	492	Yes
Cao et al.	2011	170	307	135	191	307	134	577	647	575	689	Yes
Wang et al.	2013	121	139	46	92	154	60	231	381	274	338	Yes
Jing et al.	2013	78	93	27	47	76	33	147	249	142	170	Yes
Kang et al.	2013	180	207	78	248	381	170	363	567	721	877	Yes
Pan et al.	2013	114	384	321	98	369	336	1026	612	1041	565	Yes
Zhang et al.	2013	48	52	24	46	65	30	100	148	125	157	Yes
Li et al.	2013	71	126	34	94	143	63	194	268	269	331	Yes
Zhang et al.	2014	93	102	52	93	140	67	206	288	274	326	Yes
Luo et al.	2014	64	86	44	70	128	47	174	214	222	268	Yes
Mashayekhi et al.	2015	58	80	12	41	102	7	104	196	116	184	No
Lai et al.	2016	217	368	136	211	380	140	640	802	660	802	Yes
Meng et al.	2017	182	285	104	211	324	122	493	649	568	746	Yes
Meng et al.	2017	199	298	111	386	564	215	520	696	994	1336	Yes
Xiao et al.	2017	80	70	22	86	121	49	114	230	219	293	Yes
Xiao et al.	2017	83	93	30	108	178	74	153	259	326	394	Yes
Lu et al.	2017	189	GT/GG = 288		179	GT/GG = 379						Yes

### Quantitative synthesis

A total of 23 independent case–control studies with 10166 cancer cases and 11598 controls were included to explore the potential correlation of *APE1* rs1760944 T>G polymorphism with the susceptibility of cancer [23–25,31–47]. We found that *APE1* rs1760944 T>G polymorphism conferred statistical evidence of the relationship between *APE1* rs1760944 T>G locus and a decreased risk of cancer (G vs. T: OR, 0.87; 95% CI, 0.83–0.92 *P*<0.001; GG vs. TT: OR, 0.77; 95% CI, 0.69–0.86; *P*<0.001; GG/TG vs. TT: OR, 0.83; 95% CI, 0.77–0.89, *P*<0.001 and GG vs. TT/TG: OR, 0.85; 95% CI, 0.80–0.92, *P*<0.001; Table 3).

**Table 3:** Results of the meta-analysis from different genetic models

	Number of cases/controls	G vs. T				GG vs. TT				GG/TG vs. TT				GG vs. TT/TG			
		OR (95% CI)	*P*	*I^2^*	*P* (Q-test)	OR (95% CI)	*P*	*I^2^*	*P* (Q-test)	OR(95% CI)	*P*	*I^2^*	*P* (Q-test)	OR (95% CI)	*P*	*I^2^*	*P* (Q-test)
Total	9997/11537	**0.87 (0.83–0.92)**	**<0.001**	43.9%	0.015	**0.77 (0.69–0.86)**	**<0.001**	39.4%	0.031	**0.83 (0.77–0.89)**	**<0.001**	35.8%	0.046	0.85 (0.80**–**0.92)	**<0.001**	26.8%	0.121
HWE																	
Yes	9847/11387	**0.87 (0.82–0.92)**	**<0.001**	46.4%	0.011	**0.77 (0.69–0.85)**	**<0.001**	41.1%	0.026	**0.83 (0.77–0.90)**	**<0.001**	35.1%	0.054	**0.85 (0.79–0.91)**	**<0.001**	24.4%	0.151
No		0.84 (0.60**–**1.17)	0.310	-	-	1.21 (0.44**–**3.34)	0.710	-	-	**0.60 (0.37–0.97)**	**0.038**	-	-	1.78 (0.68**–**4.64)	0.241	-	-
Ethnicity																	
Caucasians	810/824	1.00 (0.87**–**1.15)	0.994	20.0%	0.264	1.09 (0.81**–**1.48)	0.573	0.0%	0.832	0.82 (0.47**–**1.45)	0.502	75.1%	0.045	1.07 (0.86**–**1.33)	0.539	11.5%	0.288
Asians	9187/10713	**0.86 (0.82–0.91)**	**<0.001**	42.4%	0.024	**0.75 (0.67–0.84)**	**<0.001**	36.3%	0.054	**0.82 (0.76–0.89)**	**<0.001**	32.9%	0.073	**0.83 (0.78–0.90)**	**<0.001**	17.7%	0.234
Cancer type																	
Colorectal cancer	1628/1705	0.97 (0.88**–**1.07)	0.607	0.0%	0.396	0.96 (0.79**–**1.17)	0.682	0.0%	0.510	0.93 (0.80**–**1.09)	0.388	15.3%	0.307	1.00 (0.86**–**1.17)	0.983	0.0%	0.873
Lung cancer	3071/3038	**0.83 (0.78–0.90)**	**<0.001**	0.0%	0.717	**0.68 (0.59–0.79)**	**<0.001**	0.0%	0.702	**0.80 (0.72–0.90)**	**<0.001**	0.0%	0.795	**0.77 (0.68–0.87)**	**<0.001**	10.8%	0.344
Cervical cancer	1485/2128	0.93 (0.79**–**1.09)	0.361	61.4%	0.075	0.87 (0.65**–**1.17)	0.367	51.5%	0.127	0.90 (0.71**–**1.15)	0.416	62.3%	0.071	0.93 (0.78**–**1.11)	0.433	0.0%	0.438
Breast cancer	809/1194	**0.83 (0.73–0.95)**	**0.005**	4.3%	0.352	**0.75 (0.57–0.98)**	**0.034**	37.6%	0.202	**0.71 (0.59–0.86)**	**<0.001**	0.0%	0.635	1.04 (0.65**–**1.67)	0.870	62.1%	0.072
Nasopharyngeal cancer	708/858	0.89 (0.70**–**1.14)	0.355	-	-	0.71 (0.43**–**1.20)	0.204	-	-	0.84 (0.59**–**1.18)	0.316	58.5%	0.120	0.65 (0.41**–**1.03)	0.064	-	-
Osteosarcoma	378/616	**0.69 (0.57–0.83)**	**<0.001**	0.0%	0.701	**0.51 (0.35–0.75)**	**0.001**	0.0%	0.823	**0.61 (0.47–0.80)**	**<0.001**	0.0%	0.748	**0.64 (0.45–0.91)**	**0.014**	0.0%	0.867
Others	1918/1998	0.87 (0.75**–**1.02)	0.080	58.0%	0.049	0.77 (0.57**–**1.03)	0.083	54.7%	0.065	0.89 (0.78**–**1.02)	0.109	47.8%	0.105	0.87 (0.74**–**1.02)	0.079	22.3%	0.273
Source of control																	
Population-based	1160/1191	0.92 (0.73**–**1.17)	0.506	75.6%	0.043	0.83 (0.50**–**1.40)	0.494	78.5%	0.031	0.93 (0.77**–**1.13)	0.485	28.9%	0.236	0.84 (0.54**–**1.31)	0.448	80.5%	0.024
Hospital-based	8837/10346	0.87 (0.82**–**0.92)	**<0.001**	41.3%	0.028	**0.76 (0.68–0.85)**	**<0.001**	35.5%	0.059	0.82 (0.75**–**0.88)	**<0.001**	36.7%	0.048	0.85 (0.79**–**0.92)	**<0.001**	18.3%	0.226

Bold values are statistically significant (*P*< 0.05).

**Figure 2:**
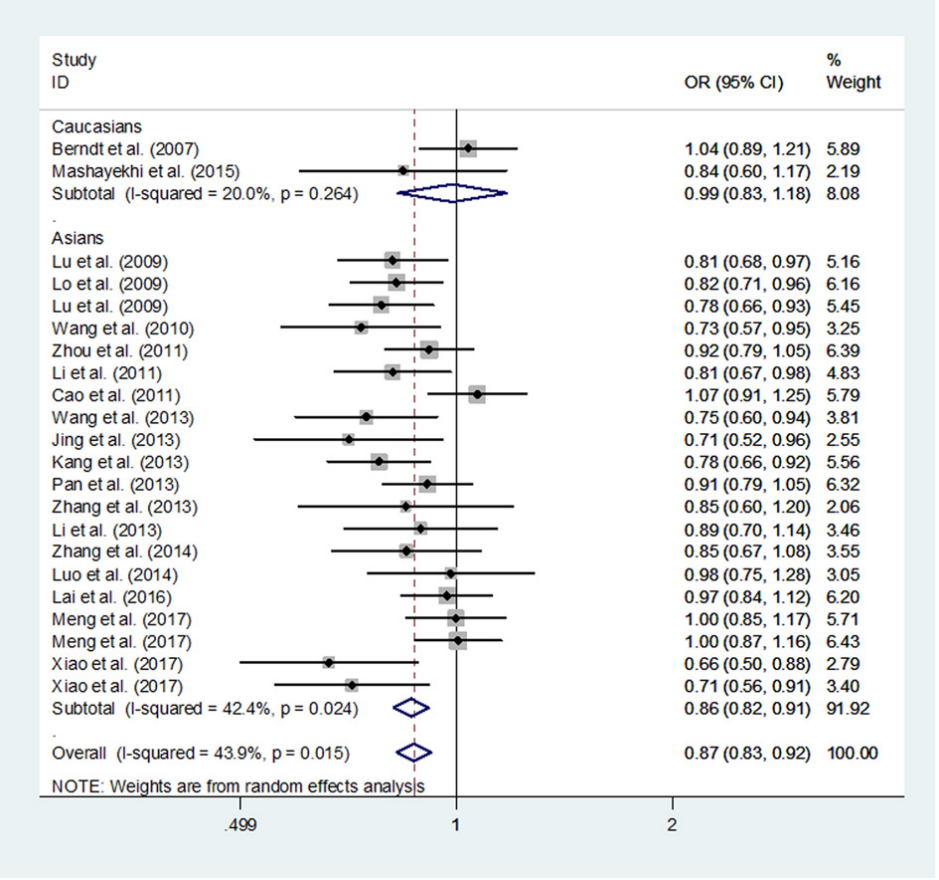
Meta-analysis for the association of cancer risk with the *APE1* rs1760944 T>G polymorphism (random-effect, allele comparing model)

When we conducted subgroup analyses according to the different populations, the findings indicated that *APE1* rs1760944 T>G polymorphism might be a protective factor for the development of cancer in Asian population (G vs. T: OR, 0.86; 95% CI, 0.82–0.91 *P*<0.001; GG vs. TT: OR, 0.75; 95% CI, 0.67–0.84; *P*<0.001; GG/TG vs. TT: OR, 0.82; 95% CI, 0.76–0.89, *P*<0.001 and GG vs. TT/TG: OR, 0.83; 95% CI, 0.78–0.90, *P*<0.001; Figure 2).

When we conducted subgroup analyses according to cancer type, the results suggested that *APE1* rs1760944 T>G polymorphism decreased the risk of lung cancer (G vs. T: OR, 0.83; 95% CI, 0.78–0.90, *P*<0.001; GG vs. TT: OR, 0.68; 95% CI, 0.59–0.79; *P*<0.001; GG/TG vs. TT: OR, 0.80; 95% CI, 0.72–0.90, *P*<0.001 and GG vs. TT/TG: OR, 0.77; 95% CI, 0.68–0.87, *P*<0.001), breast cancer (G vs. T: OR, 0.83; 95% CI, 0.73–0.95, *P*=0.005; GG vs. TT: OR, 0.75; 95% CI, 0.57–0.98; *P*=0.034 and GG/TG vs. TT: OR, 0.71; 95% CI, 0.59–0.86, *P*<0.001), and osteosarcoma (G vs. T: OR, 0.69; 95% CI, 0.57–0.83 *P*<0.001; GG vs. TT: OR, 0.51; 95% CI, 0.35–0.75; *P*=0.001; GG/TG vs. TT: OR, 0.61; 95% CI, 0.47–0.80, *P*<0.001 and GG vs. TT/TG: OR, 0.64; 95% CI, 0.45–0.91, *P*=0.014).

### Publication bias and non-parametric ‘trim-and-fill’ method

**Figure 3:**
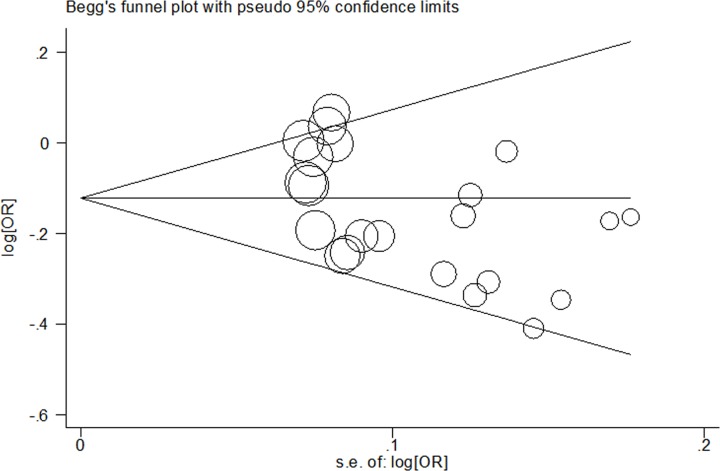
For *APE1* rs1760944 T>G polymorphism, Begg’s funnel plot analysis for publication bias (allele comparing model)

In the present study, Begg’s and Egger’s tests were used to assess the potential bias among the eligible studies. Evidence of bias was found in the present study (G vs. T: Begg’s test *P*=0.055, Egger’s test *P*=0.013; GG vs. TT: Begg’s test *P*=0.037, Egger’s test *P*=0.080; GG/TG vs. TT: Begg’s test *P*=0.051, Egger’s test *P*=0.016; GG vs. TT/TG: Begg’s test *P*=0.055, Egger’s test *P*=0.174; Figure 3).

**Figure 4:**
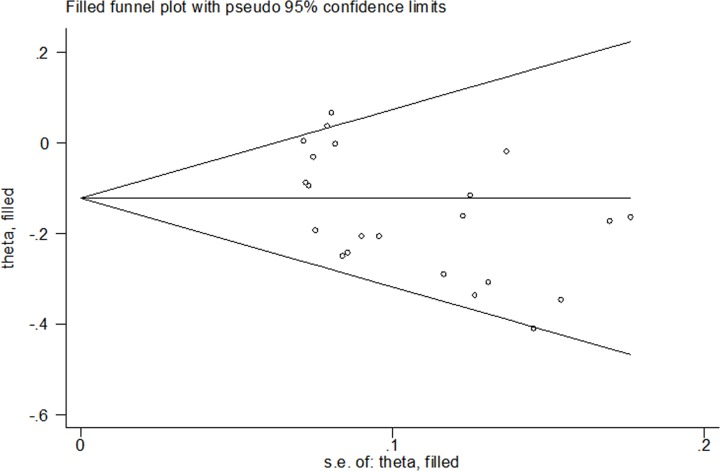
For *APE1* rs1760944 T>G polymorphism, filled funnel plot of meta-analysis (allele comparing model)

Since bias was found, we used non-parametric ‘trim-and-fill’ method to evaluate the stability of results. When we treated the publication bias, the adjusted ORs and CIs were not significantly changed (Figure 4).

### Sensitivity analysis

**Figure 5:**
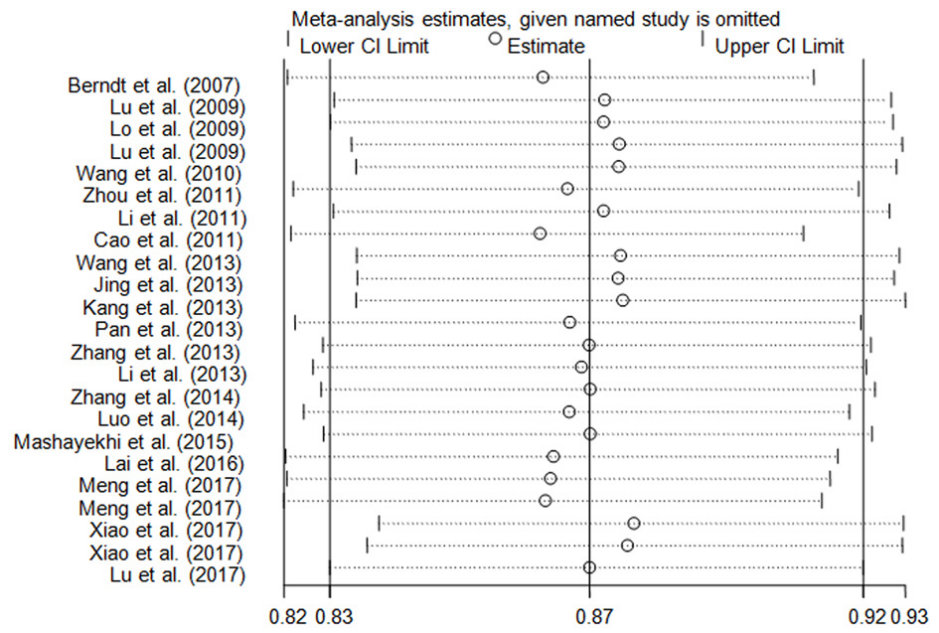
Sensitivity analysis of the influence in G vs. T genetic model (random-effects estimates)

In this meta-analysis, sensitivity analysis was conducted by one-way method, which deleted an individual case–control study one by one and re-calculated the pooled ORs and CIs. No single case–control study significantly influenced the final decision (Figure 5).

### Heterogeneity

We found significant heterogeneity in all genetic models. Considering the potential factors for heterogeneity, subgroup analysis was conducted to identify its major source. In this meta-analysis, Asians, cervical cancer and population-based studies contribute to the major sources of heterogeneity.

## Discussion

The *APE1* rs1760944 T>G has been frequently investigated due to its potential role in the development of cancer; however, the results are conflicting. To shed light on this issue, we performed an extensive meta-analysis. The results highlighted that *APE1* rs1760944 T>G polymorphism decreased the risk of cancer. Results of subgroup analyses demonstrated that this SNP still significantly modified the risk among lung cancer, breast cancer, osteosarcoma patients and Asians.

Rs1760944 T>G is a promoter SNP in the *APE1* gene and may affect the binding of transcription factors. Since 2007, a number of case–control studies were performed to assess the potential relationship of *APE1* rs1760944 T>G polymorphism with the risk of cancer, but the observations were controversial. Several investigations suggested that *APE1* rs1760944 T>G SNP decreased the susceptibility of cancer [23,24,31,32,37,38,40,42,44,46]. However, other case–control studies suggest null correlation between the *APE1* rs1760944 T>G SNP and cancer risk [25,33–36,39,41,43,45,47]. How can we obtain an extensive evaluation of the relationship between *APE1* rs1760944 T>G locus and the risk of cancer with the consistent conclusions? To our knowledge, small sample size investigation could lead to confusing findings. Thus, we carried out a meta-analysis with 23 independent case–control studies to explore the correlation between *APE1* rs1760944 T>G SNP and the susceptibility of cancer. In the included case–control studies, χ^2^ test was used to calculate the pooled ORs and CIs. In meta-analysis, we also used χ^2^ test to evaluate the relationship of *APE1* rs1760944 T>G polymorphism with cancer risk. Overall, we found that *APE1* rs1760944 T>G polymorphism decreased the risk of cancer in four genetic models. When we conducted subgroup analyses, we found that *APE1* rs1760944 T>G polymorphism decreased the risk of lung cancer, breast cancer, osteosarcoma and Asians. To the best of our knowledge, the association might be confounded by some potential bias (e.g. publication bias, heterogeneity and lack of accordance with HWE in controls). Thus, we subsequently performed subgroup analyses. The findings suggested that the *APE1* rs1760944 T>G polymorphism might be a protective effect on the development of cancer in Asians only, but not Caucasians. In the present study, one case–control study was incongruent with HWE [37]. When we deleted it and re-calculated the pooled ORs and CIs, the significant relationship was not changed. In the present study, we conducted non-parametric ‘trim-and-fill’ method to explore the potential influence of publication bias. We found that the bias of publication might not alter the findings. We also found that *APE1* rs1760944 T>G polymorphism still significantly decreased the risk of some type of cancers.

It was found that inhibition of APE1 activity might reduce cell growth of ovarian cancer [20] and pancreatic cancer [21]. In addition, Luo et al. [48] identified that a decreased APE1 activity could also significantly retard the proliferation of endothelial cells, suggesting its stimulative effect on the development of cancer. Several studies indicated that *APE1* rs1760944 G allele decreased *APE1* mRNA and protein expression levels [23,24]. Additionally, Lu et al. [24] reported that *APE1* rs1760944 G allele was associated with a decreased level of APE1 mRNA by reducing the binding affinity of some transcription factors. Although the pathway of the relationship between *APE1* rs1760944 T>G and cancer risk has been not confirmed, it is speculated that this SNP may alter the susceptibility of cancer through the mechanism mentioned above. All observations and speculations should be verified with new molecular studies.

Some limitations of the current analysis should be noted. First, in this meta-analysis, only published literature was eligible and included, and some presumable unpublished studies might be neglected and discarded. Second, heterogeneity and publication bias were apparent, which could distort the pooled results. Our findings should be interpreted with cautions. Third, for lack of sufficient data (e.g., smoking, drinking, age, sex and vegetable and fruit intake and other environmental factors), we only conducted a crude assessment. Finally, only *APE1* rs1760944 T>G polymorphism was included to assess the association with the risk of cancer; other functional loci in *APE1* gene should not been ignored.

In summary, this updated meta-analysis highlights that the *APE1* rs1760944 T>G polymorphism may play a protective role in the development of cancer. Further studies in different race are needed to confirm or refute our findings.

## Abbreviations

APE1: apurinic/apyrimidinic endonuclease 1
CI: confidence interval
HWE: Hardy–Weinberg equilibrium
OR: odds ratio
SNP: single-nucleotide polymorphism
